# High Frequency of *Chlamydia trachomatis* Mixed Infections Detected by Microarray Assay in South American Samples

**DOI:** 10.1371/journal.pone.0153511

**Published:** 2016-04-15

**Authors:** Lucía Gallo Vaulet, Carolina Entrocassi, Ana I. Portu, Erica Castro, Susana Di Bartolomeo, Anke Ruettger, Konrad Sachse, Marcelo Rodriguez Fermepin

**Affiliations:** 1 Universidad de Buenos Aires. Cátedra de Microbiología Clínica, Facultad de Farmacia y Bioquímica, Buenos Aires, Argentina; 2 Facultad de Medicina, Universidad de San Sebastián, Concepción, Chile; 3 Sección Bioquímica Microbiológica, Servicio de Bioquímica, Hospital Nacional Profesor A. Posadas, Buenos Aires, Argentina; 4 Institute of Molecular Pathogenesis, Friedrich-Loeffler-Institut (Federal Research Institute for Animal Health), Jena, Germany; Oregon State University, UNITED STATES

## Abstract

*Chlamydia trachomatis* is one of the most common sexually transmitted infections worldwide. Based on sequence variation in the *omp*A gene encoding the major outer membrane protein, the genotyping scheme distinguishes 17 recognized genotypes, i.e. A, B, Ba, C, D, Da, E, F, G, H, I, Ia, J, K, L1, L2, and L3. Genotyping is an important tool for epidemiological tracking of *C*. *trachomatis* infections, including the revelation of transmission pathways and association with tissue tropism and pathogenicity. Moreover, genotyping can be useful for clinicians to establish the correct treatment when LGV strains are detected. Recently a microarray assay was described that offers several advantages, such as rapidity, ease of standardization and detection of mixed infections. The aim of this study was to evaluate the performance of the DNA microarray-based assay for *C*. *trachomatis* genotyping of clinical samples already typed by PCR-RFLP from South America. The agreement between both typing techniques was 90.05% and the overall genotype distribution obtained with both techniques was similar. Detection of mixed-genotype infections was significantly higher using the microarray assay (8.4% of cases) compared to PCR-RFLP (0.5%). Among 178 samples, the microarray assay identified 10 *omp*A genotypes, i.e. D, Da, E, F, G, H, I, J, K and L2. The most predominant type was genotype E, followed by D and F.

## Introduction

*Chlamydia (C*.*) trachomatis* is one of the most common sexually transmitted infections worldwide. If left untreated, serious sequelae may arise, such as ectopic pregnancy and infertility in women and epididymitis and proctitis in men [1].

*C*. *trachomatis* is classified into 17 genotypes according to the sequence variation within the *omp*A gene, which encodes the major outer membrane protein. The well-known genotypes A, B, Ba, C, D, Da, E, F, G, H, I, Ia, J, K, L1, L2, and L3 can also be grouped according to pathology from which they were isolated. While genotypes A to C are commonly associated with trachoma, genotypes D to K primarily cause urogenital infections and genotypes L1 to L3are *lymphogranuloma venereum* agents, a more invasive sexually transmitted disease [1–4].

Genotyping is an important tool to understand the epidemiology of *C*. *trachomatis* and may also be useful for elucidating transmission pathways and associations with different tissue tropisms and pathogenicity [5–8]. Historically, one of the most used techniques for genotyping of *C*. *trachomatis* was the *omp*A gene PCR-restriction fragment length polymorphism (RFLP) analysis. However, the method has some weak points, such as the emergence of atypical restriction patterns due to mixed-genotype infections, artifacts from the enzymatic digestion, and ambiguities due to polymorphisms in the restriction sites or *omp*A recombinants. The resolution of such atypical patterns requires cumbersome and time-consuming analysis and/or additional runs with different restriction enzymes. Moreover, this methodology might not properly perform when it comes to detection of known or unknown varieties of genotypes.

In view of these limitations, *C*. *trachomatis* genotyping by PCR-RFLP has been replaced with more sophisticated methodologies, such as *omp*A gene sequencing [9, 10], reverse dot blot [11, 12], several real-time PCR protocols using fluorescent probes [13, 14], and DNA microarray assay [15]. There is still no agreement on which methodology is the most accurate to be regarded as “gold standard”. Moreover, most of these technologies are time consuming and expensive, which precludes their routine use in Latin American diagnostic facilities.

The microarray typing assay has emerged as a promising alternative to conventional typing technology in microbiology [16–18]. While most of the commercially available microarray devices are still expensive and out of reach for most clinical diagnostic laboratories, the ArrayTube^TM^ and ArrayStrip^TM^ platforms have proven to be suitable for routine diagnostic applications [19]. In the specific field of chlamydiae, microarray technology has been applied for species identification of the family *Chlamydiaceae* [20], and genotyping of *Chlamydia psittaci* [21]. Among the main advantages of the recently described microarray typing assay for *C*. *trachomatis* include its rapidity (results available within one working day), the possibility to efficiently detect mixed infections, and objectivity in the interpretation of the data [15].

While this technology can be integrated into a clinical diagnostic setting, it was designed and validated with reference strains and with European clinical samples and it was not clear whether this system would also adequately detect the genotypic variants prevailing in a geographically distant region.

The aim of this study was to evaluate the performance of the DNA microarray-based assay for *C*. *trachomatis* genotyping on clinical samples obtained from two hospitals located in Buenos Aires, Argentina and clinical samples obtained in a *C*. *trachomatis* prevalence study in Concepción, Chile

## Materials and Methods

### Clinical samples

A total of 182 *C*. *trachomatis*-positive samples from two hospitals located in Buenos Aires obtained between 2005 and 2007 and from a *C*. *trachomatis* prevalence study conducted in Concepcion City, Chile, in 2005, were included in this study. *C*. *trachomatis* was detected in all samples by *omp*A nested PCR and genotyped by RFLP analysis as previously described [22]. Fifty-four samples were collected at the Hospital de Clínicas “Jose de San Martin” which belongs to the Universidad de Buenos Aires and 87 samples at the National Hospital “Prof. A. Posadas”. The Chilean Chlamydia-positive samples were from 41 women. More details of the samples are given in Table 1.

**Table 1:** Type and origin of samples included in this study.

Type of sample	University Hospital“José de SanMartín”	National Hospital“Prof. A. Posadas”	ConcepciónChile	Total
Cervical swab	35	33	41	109
Male Urethral swab	19	18	-	37
Conjunctival swab from neonatal conjunctivitis cases	-	36	-	36
Total	54	87	41	182

### Ethics statement

All samples used in this study were re-coded in order to anonymize patient records/information prior to analysis. Therefore individuals could not be matched with their samples and their epidemiological and clinical data. Samples and epidemiological data were collected for diagnostic purpose under standards of care protocols for sexually transmitted infections in each location. Informed consent, as approved by our institutional Ethics Committee, was not required and only requested to parents for newborn samples as stipulated by the National Hospital “Prof. A. Posadas” Ethics Committee. Neither additional samples nor personal data were requested for this study. Moreover, genetic analysis was only performed on bacterial DNA isolates. The study was conducted according to the principles expressed in the Declaration of Helsinki and was approved by the Ethics Committee of the Facultad de Farmacia y Bioquímica, Universidad de Buenos Aires.

### DNA extraction

DNA was extracted from the samples using the QIAamp DNA minikit QIAgen (Qiagen, Hilden, Germany) according to the manufacturer’s instructions.

### Genotyping of *C*. *trachomatis* by microarray assay

Chlamydial DNA from clinical samples was amplified and biotin labeled using a multiplex PCR protocol, which included five *omp*A primers covering variable domains 1, 2 and 4 as previously described [15].

ArrayStrip^TM^ units consisting of 8 connected plastic vessels in microtiter format, each carrying a microarray chip, were used. The AS hybridization reactions were performed using the Hybridization Kit (Alere Technologies GmbH, Jena, Germany) following the instructions of the manufacturer as previously described [15]. Hybridization signals were processed using the Iconoclust software, version 3.3 (Alere). The Pattern Match algorithm integrated in the Partisan Array LIMS database software system (Alere) was used for automatic assignment and genotype identification as described elsewhere [15].

The matching score (MS), which represents a measure of the overall similarity between two hybridization patterns, served as the guiding parameter for quality assessment of the hybridization data and final genotype assignment. All hybridization experiments yielding MS<10 allowed direct acceptance of the automatically determined genotype, whereas values greater than 10 suggested the presence of additional hybridization signals probably due to more than one genotype in the sample. In order to confirm the mixed infection, we checked the automatic assignment by visually comparing the hybridization pattern of the sample with reference experiments from the database representing individual genotype reference strains. In the case of MS values greater than 20, the experiment was repeated as reliable genotyping was not possible.

### *C*. *trachomatis omp*A amplification and sequencing

*C*. *trachomatis omp*A gene of samples containing more than one genotype or conflicting results between PCR-RFLP and microarray assay were amplified as previously described [22]. The PCR products were sequenced on an automated capillary DNA sequencing system ABI3730XL (Applied Biosystems, Foster City, CA). Sequencing was performed by Macrogen Inc, Korea. Sequences were assembled, edited, and compared to chlamydia GenBank sequences for identification.

## Results

### Clinical samples

Of the 182 samples examined, 181 (99.4%) were successfully genotyped by *omp*A PCR-RFLP and 178 by microarray assay (97.8%).

In one sample, genotyping failed with both techniques. In three samples, the microarray assay was not successful. In another sample, there was no agreement between the genotype obtained with both techniques.

### Distribution of *omp*A genotypes

The spectrum of genotypes identified by each genotyping technique is shown in Table 2. Among the 181 samples, we identified the following 8 *omp*A genotypes by utilizing PCR-FRLP: genotypes D, E, F, G, H, I, J, and K. On the other hand, among the 178 samples analyzed by microarray assay 10 *omp*A genotypes D, Da, E, F, G, H, I, J, K and L2 were identified. Regardless of the typing method used, the most predominant genotype found was genotype E (50.3% by PCR-RFLP and 55.6% by microarray assay), followed by D (including Da) and F using PCR-RFLP (13.2% and 11.6% respectively) and F and G using microarray assay (13.4% and 9.5% respectively). Genotypes Da and L2 were only detected using microarray assay. Overall, the genotype distribution obtained was similar using both techniques. The distribution of *C*. *trachomatis* genotypes in each hospital and type of sample is given in Table 3.

**Table 2:** *C*. *trachomatis* genotype distribution according to genotyping technique.

Genotype	Genotyping technique	
	PCR-RFLP^a^	Microarray assay^a^
	n	n
D	24	15
Da	0	10
E	91	99
F	21	24
G	17	17
H	6	4
I	7	7
J	13	14
K	3	3
L2	0	1
Total genotypes detected	182	194
Total samples successfully genotyped	181	178

^a^Genotypes I, Ia, J and Ja are indistinguishable using both methodologies.

**Table 3:** Distribution of *C*. *trachomatis* genotypes according to type of sample.

Origin of sample	Type of sample	Genotype	Genotyping technique	
			PCR-RFLP	Microarray
			n	n
Buenos AiresArgentinaUniversity Hospitaln = 54	Urogenital	D	**9**	**2**
		Da	**0**	**6**
		E	**24**	**28**
		F	7	7
		G	3	3
		H	**3**	**2**
		I^a^	4	4
		J^b^	3	3
		K	1	1
		L2	0	0
	Urogenital n = 50	D	**8**	**7**
		Da	**0**	**1**
Buenos Aires Argentina National Hopsital n = 86		E	**24**	**26**
		F	**9**	**10**
		G	4	4
		H	2	2
		I^a^	2	2
		J^b^	2	2
		K	0	0
		L2	**0**	**1**
	Neonatal n = 36	D	**3**	**2**
		Da	**0**	**1**
		E	**25**	**26**
		F	**3**	**5**
		G	3	3
		H	0	0
		I^a^	0	0
		J^b^	**0**	**1**
		K	2	2
		L2	0	0
Concepción Chilen = 41	Urogenital	D	4	4
		Da	0	2
		E	**18**	**19**
		F	2	2
		G	7	7
		H	**1**	**0**
		I^a^	1	1
		J^b^	8	8
		K	0	0
		L2	0	0

^a^Includes I and Ia.

^b^Includes J and Ja.

### Detection of mixed infections

The number of genotypes detected using microarray technology was higher than that detected by PCR-RFLP (Table 2), due to detection of a larger number of mixed infections. Out of the 178 samples producing conclusive results by microarray, 15 samples were identified that contained more than one *omp*A genotype (8.4%). In the University Hospital and Chilean group of samples, we detected 7.7% and 7.5% of mixed infections respectively, whereas the samples from the National Hospital had a proportion of 9.4% of mixed infections. In 13 of these 15 samples, the mixed infection included genotype E. Female samples contained 9.5% of mixed infections, followed by conjunctival samples (8.3%) and male samples (5.5%).

### Comparison between PCR-RFLP and microarray genotyping of *C*. *trachomatis*

The overall agreement between the genotypes determined by PCR-RFLP and DNA microarray over samples genotyped was 90.05% (163/181). We excluded a female urogenital sample obtained at the National Hospital that presented no hybridization signal and could not be genotyped by either microarray orPCR-RFLP, although *omp*A was detectable by two different PCR reactions and *omp*A sequencing could not be performed due to scarce DNA material left.

Discrepant results were found in three female urogenital samples (two from the University Hospital and one from Concepción, Chile) that were typeable by PCR-RFLP, but yielded inconclusive microarray data (two genotypes H and one genotype D). In only one of these samples, *omp*A gene could be sequenced and rendered genotype H.

Another discrepancy between both methods was observed in a male urogenital sample obtained at the University Hospital, where genotype E was detected by PCR-RFLP vs. genotype D by microarray analysis. Unfortunately, *omp*A gene could not be sequenced due to low DNA content. However, the results had been confirmed in repeated independent experiments on the original sample material.

Moreover, microarray analysis revealed the presence of 15 more genotypes that were part of mixed infections. These genotypes were not detected by PCR-RFLP. All in all, PCR-RFLP typing detected only 92.3% of all genotypes present and only 1 out of 15 (6.67%) cases of mixed genotype infection.

Composition and origin of samples containing more than one *omp*A genotype and conflicting result are given in Table 4.

**Table 4:** Characteristic of samples with mixed *omp*A genotype or conflicting results between genotyping methodologies.

Origin of samples	Sample ID	Type of sample	Genotyping technique		
			*omp*A Sequence	PCR-RFLP	Microarray
UniversityHospital	G6348	Male urethral swab	H	H	H+E
	G6513	Male urethral swab	N/A	F	F+E
	G6509	Cervical swab	F	F	F+E
	G6484	Cervical swab	Ia	I	I+E
	G6482	Cervical swab	H	H	Not conclusive
	G6477	Cervical swab	N/A	D	Not conclusive
	G6538	Male urethral swab	N/A	E	D
ConcepciónChile	G6476	Cervical swab	Mix^¥^	E	E+Da
	G6472	Cervical swab	D	D	D+Da
	G6495	Cervical swab	Ja	J	J+E
	G6441	Cervical swab	N/A	H	Not conclusive
National Hospital	G6383	Cervical swab	J	J	J+E
	G6399	Cervical swab	Mix^¥^	E+F	E+F
	G6411	Cervical swab	D	D	D+L2
	G6410	Cervical swab	Mix^¥^	E	F+E
	G6405	Cervical swab	Mix^¥^	J	E+J
	G6387	Cervical swab	N/A	Not conclusive	Not conclusive
	G6400	Conjunctivae swab	E	E	F+E
	G6401	Conjunctivae swab	Mix^¥^	E	J+E+F
	G6391	Conjunctivae swab	F	F	F+E

N/A: not available due to low DNA content or no sample left to perform analysis.

^¥^not analyzable due to mixed genotype infection.

## Discussion

While the discrepancy between the findings of PCR-RFLP and microarray *omp*A genotyping appeared to be considerable at first glance (Table 2), the differences can be explained by the proportion of dual genotype infections and the identification of genotype Da using the microarray assay. The present PCR-RFLP assay is not capable of efficiently discriminate between genotypes D and Da, so that when genotype D is detected by RFLP might as well be genotype Da. Taking this into account, the agreement between both typing techniques was 90.05% (163/181), and the overall genotype distribution obtained with both techniques was similar.

The major advantage of the microarray technique over PCR-RFLP was the detection of mixed infections involving more than one genotype of *C*. *trachomatis*. Those can be recognized in the microarray assay as the superposition of two or more hybridization patterns rising the MS to an unusually high value. In this study, we identified only one mixed genotype infection (0.5%) using PCR-RFLP vs. 8.4% using the microarray assay.

Even though the PCR-RFLP system was capable of identifying mixed genotype infection in a single sample, it is understood that this methodology underestimates the number of genotypes present in a given clinical sample because the most abundant genotype would be favored during PCR amplification and other genotypes present may remain undetected or present light bands in RFLP analysis that could easily be dismissed as background [10]. Moreover, it is possible to detect differences in amplification efficiency for each genotype in different PCR protocols; therefore it is necessary to establish the detection limit for each individual genotype and for each amplification-genotyping system. When the validation study of the microarray assay was conducted, mixtures of different proportions of genotypes E and F or E and D were clearly identified, on condition that their proportions ranged between 1:1 and 5:1 [15], and the performance with clinical material seemed not to be impaired. In this study, all samples containing more than one *C*. *trachomatis* genotype were subjected to *omp*A sequencing and in 5 out of 15 cases we detected mix *omp*A sequences present. Differences observed between microarray assay and sequencing could also be influenced by the predominant genotype in the mixture. When we observed in detail the hybridization patterns of the 10 cases of microarray mixed genotype samples (not detected by RFLP or *omp*A sequencing) we could observe low signal intensities for the second or third genotype. Genotyping *C*. *trachomatis* by sequencing *omp*A is a very accurate method to determine sequence variation from reference strains and is regarded as gold standard for typing although it has limitations when trying to resolve mixed *omp*A genotypes present in the same sample. This methodology, compared to microarray assay, requires more time per assay and experience to analyze the sequence data obtained. These limitations are absent when using microarray, but in terms of costs both assays are still expensive for Latin American routine clinical laboratories.

A promising new methodology that allows genome-scale direct sequencing, using a multiplexed micro-droplet PCR enrichment technology was recently published. This technique could also clearly identify *C*. *trachomatis* strains in single and mixed infections, but needs further validation with clinical samples [23].

Mixed infections with two or more genotypes of *C*. *trachomatis* are not a rare event and seem to provide an opportunity for genetic recombination between strains of different genotypes [24]. The proportion of mixed infections detected in different studies ranged between 1% [25], 2.4% [26], 3% [27], 8.7% [28], and 13% [29]. Also, the composition of these mixed infections varied from study to study. In our study, 86.7% of mixed infections included the detection of genotype E but, on the other hand, Xiong et al. detected only 2.62% of mixed infections containing genotype E using a reverse line blot hybridization assay [29]. Likewise, in our study, female samples presented more mixed infections than male samples, but Gharsallah et al. using a reverse hybridization assay as well; found an association of mixed infections with male samples [30]. These variations may be due to the choice of population in the study, as well as the detection method and the genotyping assay.

Regarding the sample that showed discordant results between methodologies, this could be due to a local variation of *C*. *trachomatis* genotype not represented on the current version of the microarray. Furthermore, the inconclusive genotyping results of the sample from the National Hospital with both methodologies may be due to a new autochthonous variant of *C*. *trachomatis omp*A that is not covered by the microarray. Also, another species of *Chlamydia* spp. could have been involved since there are reports of *C*. *abortus* in genital samples [31] and mixed infections with different *Chlamydia* spp. in conjunctival samples from individuals with trachoma [32].

The characterization of *C*. *trachomatis* genotypes in a sample could help differentiate between treatment failures and possible re-infection. It is also a useful tool for epidemiological studies because it may allow the reconstruction of sexual networks and routes of transmission. Genotyping is also useful to study the associations of different genotypes with different tissue tropism and their pathogenicity. Moreover, the identification of *lymphogranuloma venereum* isolates is essential to establish the appropriate treatment course. According to the latest CDC Sexually Transmitted Diseases Treatment Guidelines, 21 days of macrolide treatment course is recommended for LGV strains, while for non-LGV strains a single dose of Azithromycin or 7 days of oral Doxycycline is recommended [33].

In summary, the correct identification of *C*. *trachomatis omp*A genotype is still an essential prerequisite for the characterization of isolates, not only to conduct epidemiological studies but also to determine effective treatment. Accordingly, the misidentification of the genotypes present in clinical samples could confound patients follow up after treatment, hinder contact tracing studies and impact on correct treatment course when an LGV genotype is not detected. In this study, we found general agreement in the genotyping results obtained by PCR-RFLP and microarray assay. Importantly, the microarray assay had higher resolution for the detection of mixed infections in this set of samples from South America. Since the occurrence of mixed infections is frequent and this information has clinical and epidemiological relevance, genotyping of *C*. *trachomatis* should be conducted using methods of adequate performance.

## Supporting Information

S1 Appendix Microarray assay results for samples with mix*C*. *trachomatis* genotypes. (PPTX)

S1 Table *C*. *trachomatis omp*A sequencing results. (XLSX)
