# Supplementary material for: High Frequency of Chlamydia trachomatis Mixed Infections Detected by Microarray Assay in South American Samples
Source: PLoS One. 2016 Apr 15;11(4):e0153511. doi: 10.1371/journal.pone.0153511 (PMC4833370; doi:10.1371/journal.pone.0153511)
Supplement: S1 Appendix — (PPTX) [file pone.0153511.s001.pptx]

## Slide 1
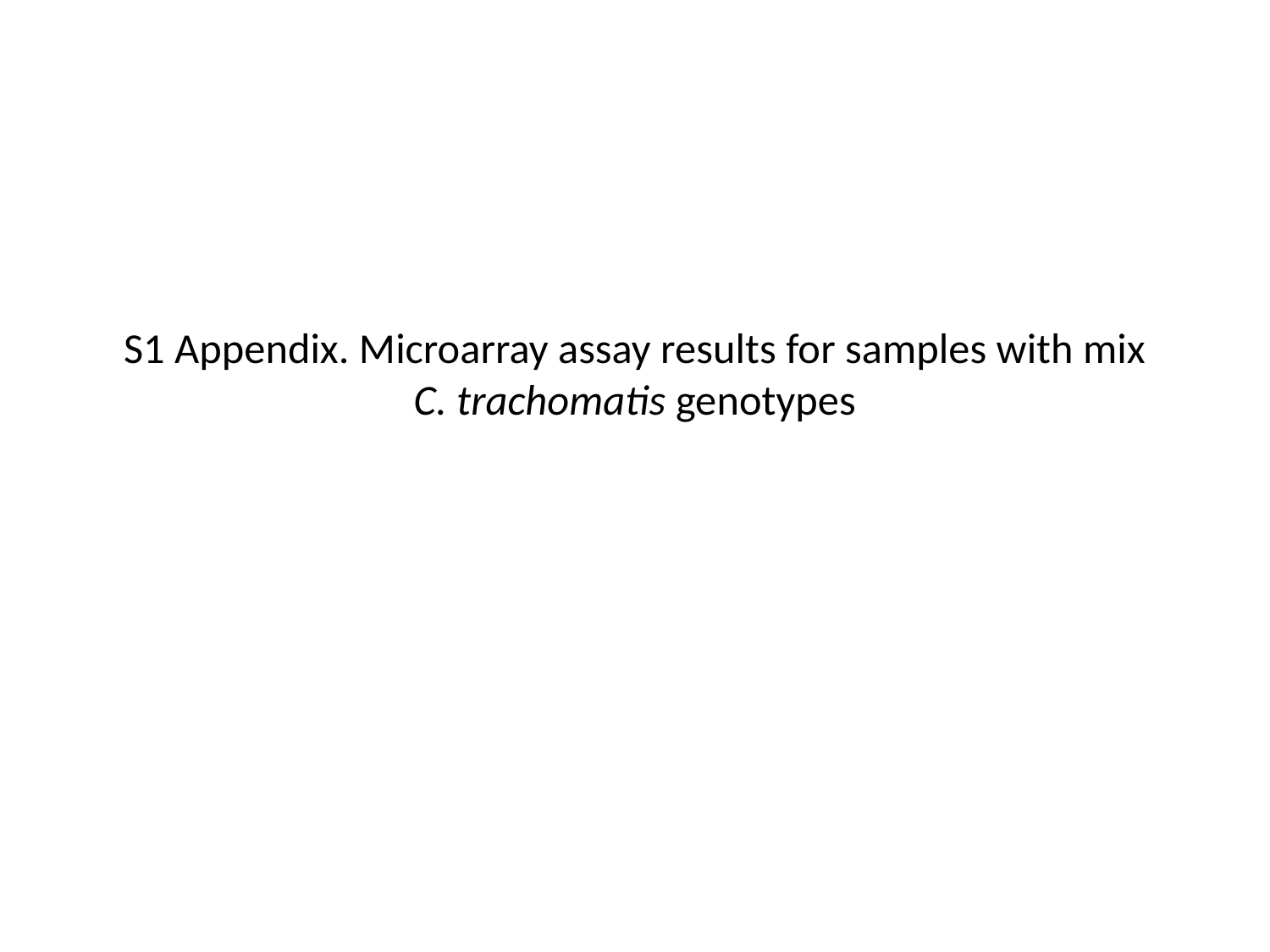

# S1 Appendix. Microarray assay results for samples with mix C. trachomatis genotypes

## Slide 2
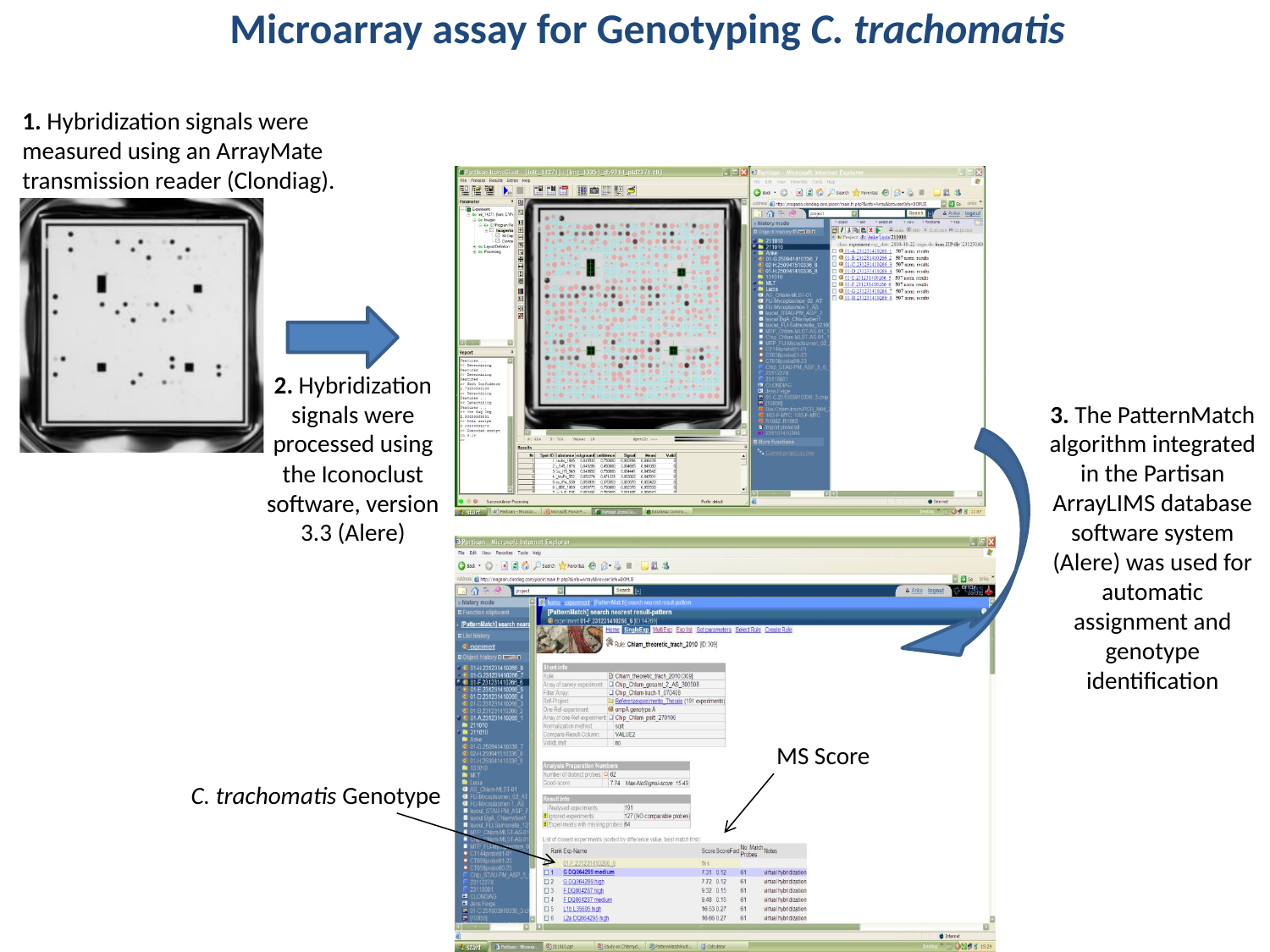

Microarray assay for Genotyping C. trachomatis
1. Hybridization signals were measured using an ArrayMate transmission reader (Clondiag).
2. Hybridization signals were processed using the Iconoclust software, version 3.3 (Alere)
3. The PatternMatch algorithm integrated in the Partisan ArrayLIMS database software system (Alere) was used for automatic assignment and genotype identification
MS Score
C. trachomatis Genotype

## Slide 3
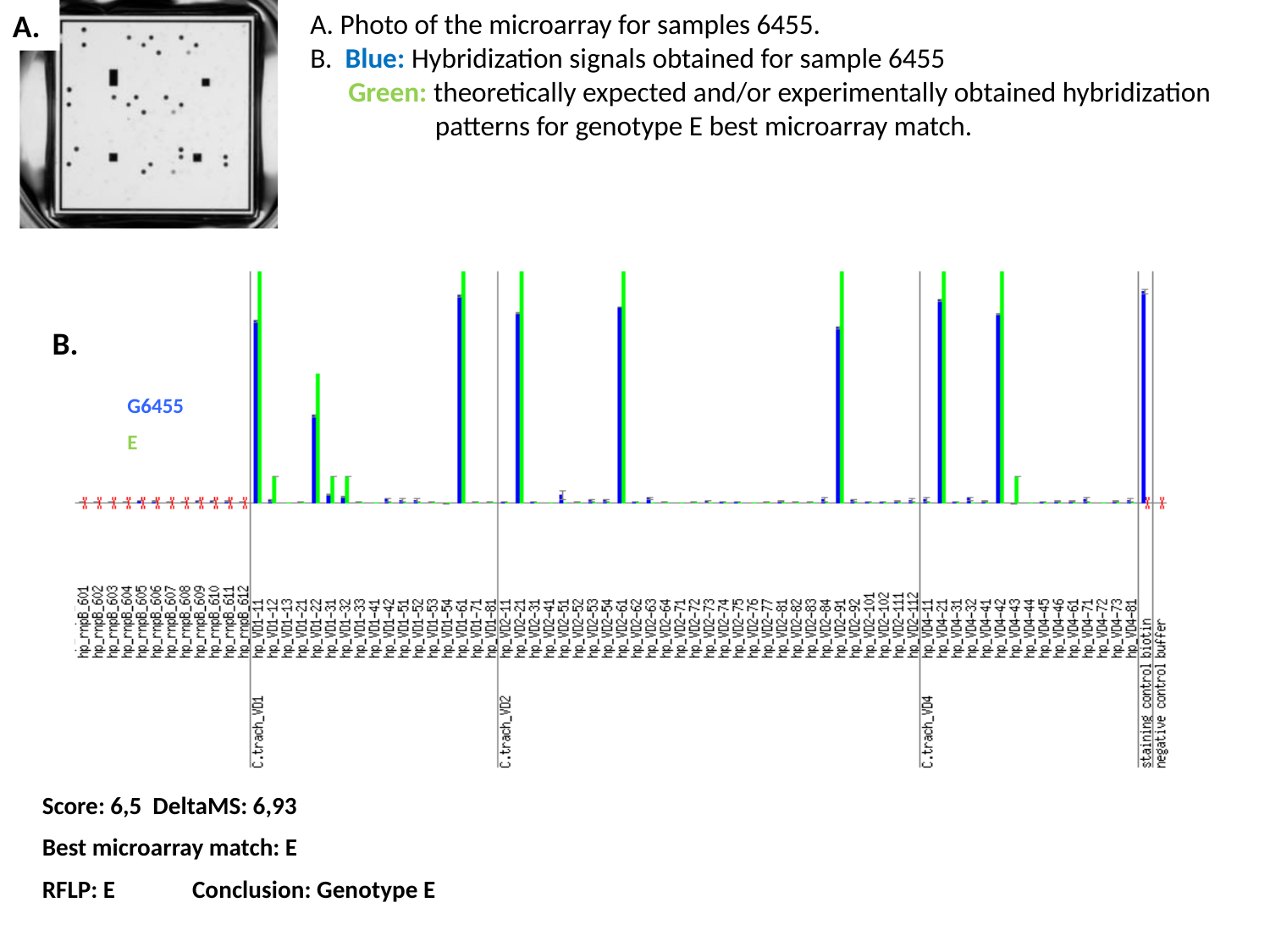

A.
A. Photo of the microarray for samples 6455.
B. Blue: Hybridization signals obtained for sample 6455
 Green: theoretically expected and/or experimentally obtained hybridization patterns for genotype E best microarray match.
B.
G6455
E
Score: 6,5 DeltaMS: 6,93
Best microarray match: E
RFLP: E Conclusion: Genotype E

## Slide 4
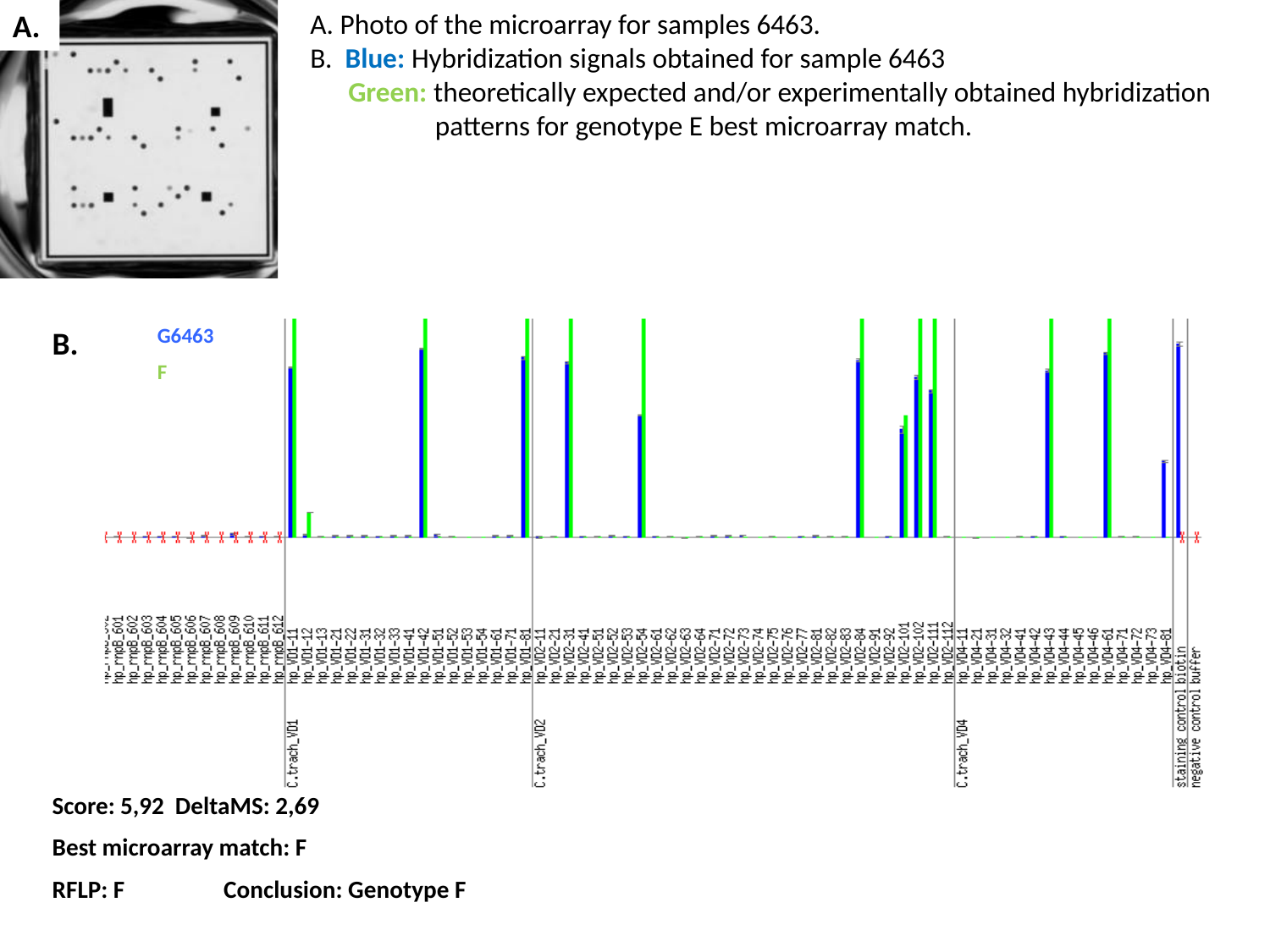

A.
A. Photo of the microarray for samples 6463.
B. Blue: Hybridization signals obtained for sample 6463
 Green: theoretically expected and/or experimentally obtained hybridization patterns for genotype E best microarray match.
G6463
F
B.
Score: 5,92 DeltaMS: 2,69
Best microarray match: F
RFLP: F Conclusion: Genotype F

## Slide 5
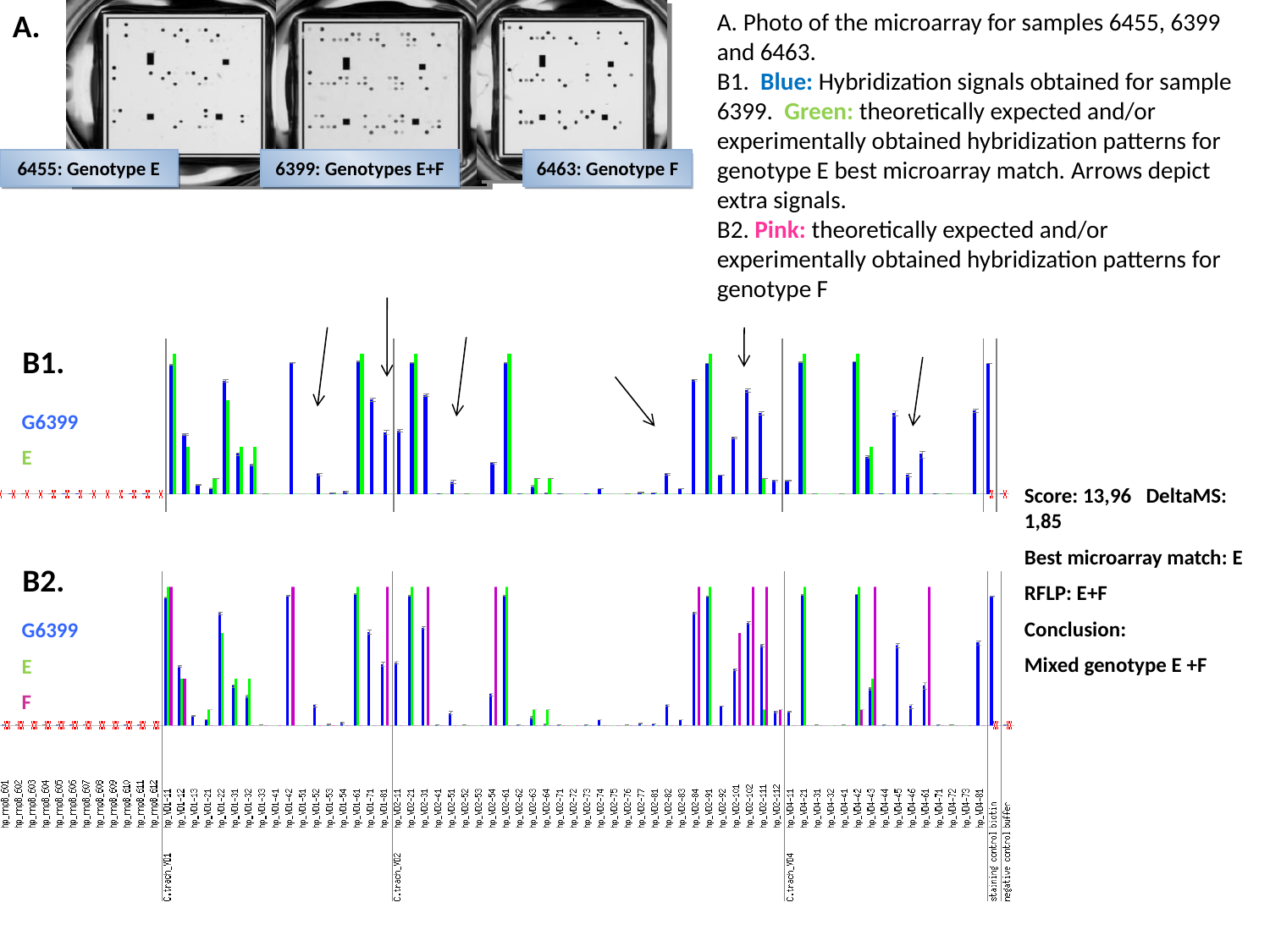

A.
6455: Genotype E
6399: Genotypes E+F
6463: Genotype F
A. Photo of the microarray for samples 6455, 6399 and 6463.
B1. Blue: Hybridization signals obtained for sample 6399. Green: theoretically expected and/or experimentally obtained hybridization patterns for genotype E best microarray match. Arrows depict extra signals.
B2. Pink: theoretically expected and/or experimentally obtained hybridization patterns for genotype F
G6399
E
G6399
E
F
B1.
Score: 13,96 DeltaMS: 1,85
Best microarray match: E
RFLP: E+F
Conclusion:
Mixed genotype E +F
B2.

## Slide 6
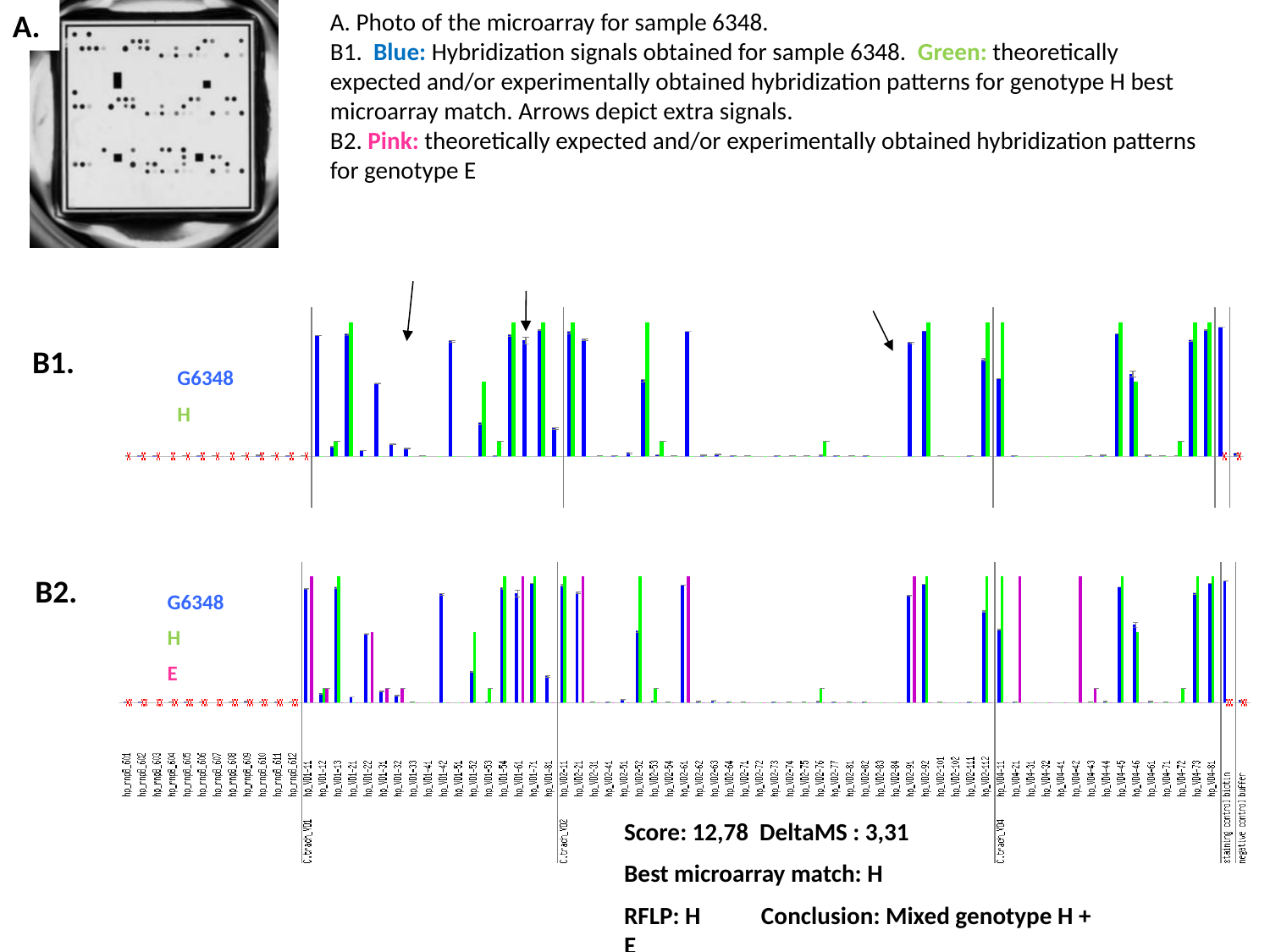

A.
A. Photo of the microarray for sample 6348.
B1. Blue: Hybridization signals obtained for sample 6348. Green: theoretically expected and/or experimentally obtained hybridization patterns for genotype H best microarray match. Arrows depict extra signals.
B2. Pink: theoretically expected and/or experimentally obtained hybridization patterns for genotype E
G6348
H
G6348
H
E
B1.
B2.
Score: 12,78 DeltaMS : 3,31
Best microarray match: H
RFLP: H Conclusion: Mixed genotype H + E

## Slide 7
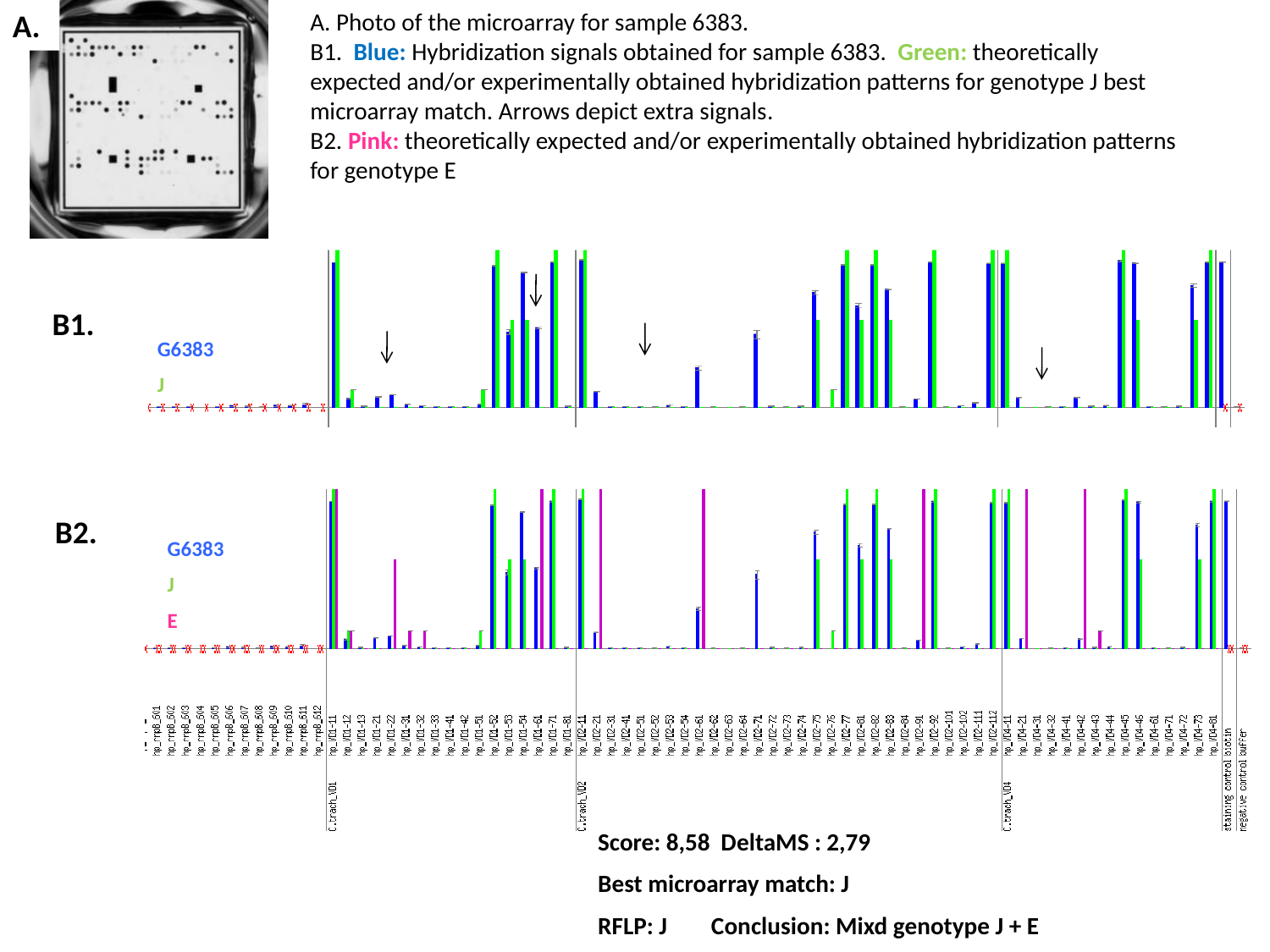

A.
A. Photo of the microarray for sample 6383.
B1. Blue: Hybridization signals obtained for sample 6383. Green: theoretically expected and/or experimentally obtained hybridization patterns for genotype J best microarray match. Arrows depict extra signals.
B2. Pink: theoretically expected and/or experimentally obtained hybridization patterns for genotype E
G6383
J
G6383
J
E
B1.
B2.
Score: 8,58 DeltaMS : 2,79
Best microarray match: J
RFLP: J Conclusion: Mixd genotype J + E

## Slide 8
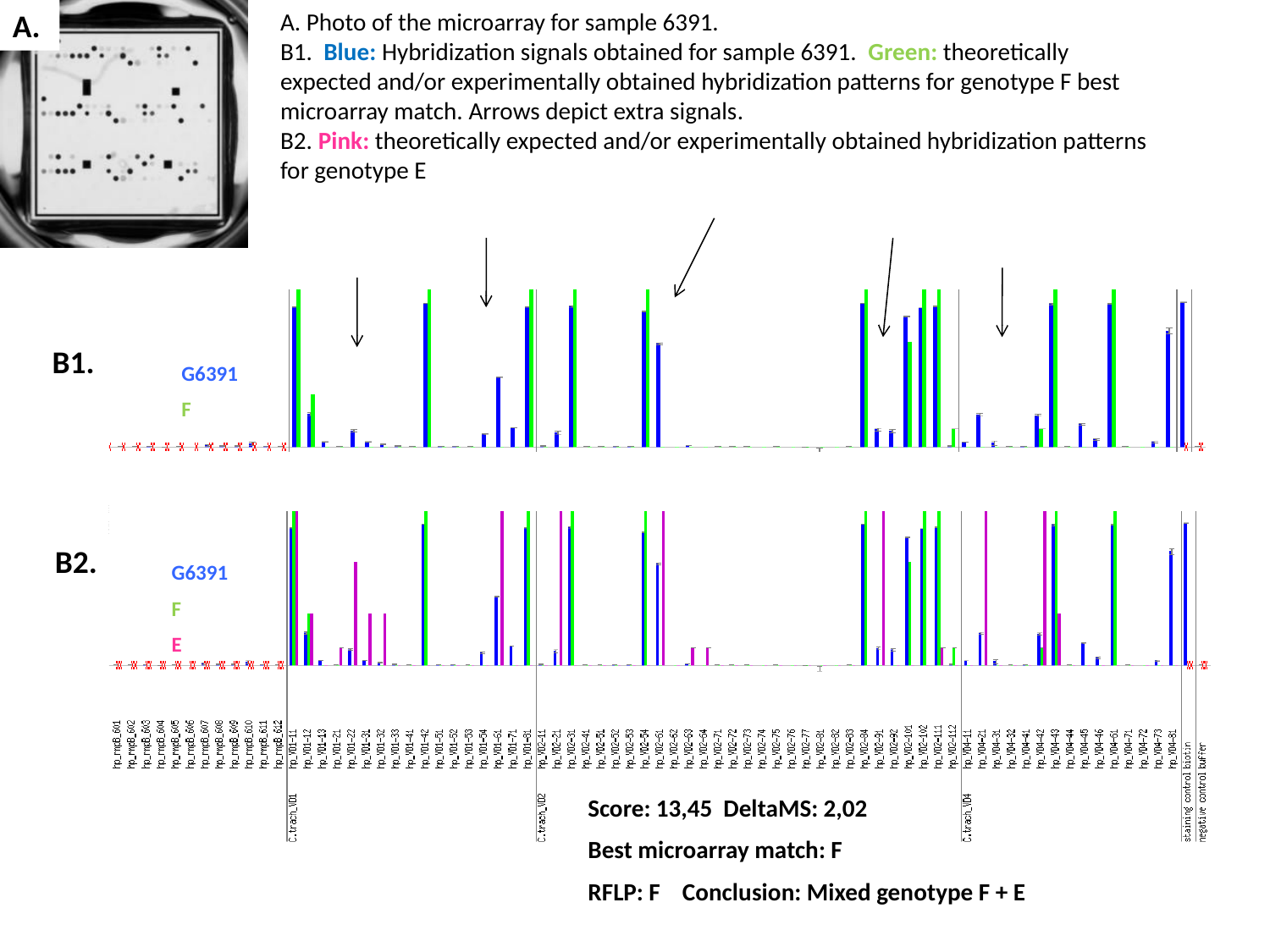

A.
A. Photo of the microarray for sample 6391.
B1. Blue: Hybridization signals obtained for sample 6391. Green: theoretically expected and/or experimentally obtained hybridization patterns for genotype F best microarray match. Arrows depict extra signals.
B2. Pink: theoretically expected and/or experimentally obtained hybridization patterns for genotype E
G6391
F
G6391
F
E
B1.
B2.
Score: 13,45 DeltaMS: 2,02
Best microarray match: F
RFLP: F Conclusion: Mixed genotype F + E

## Slide 9
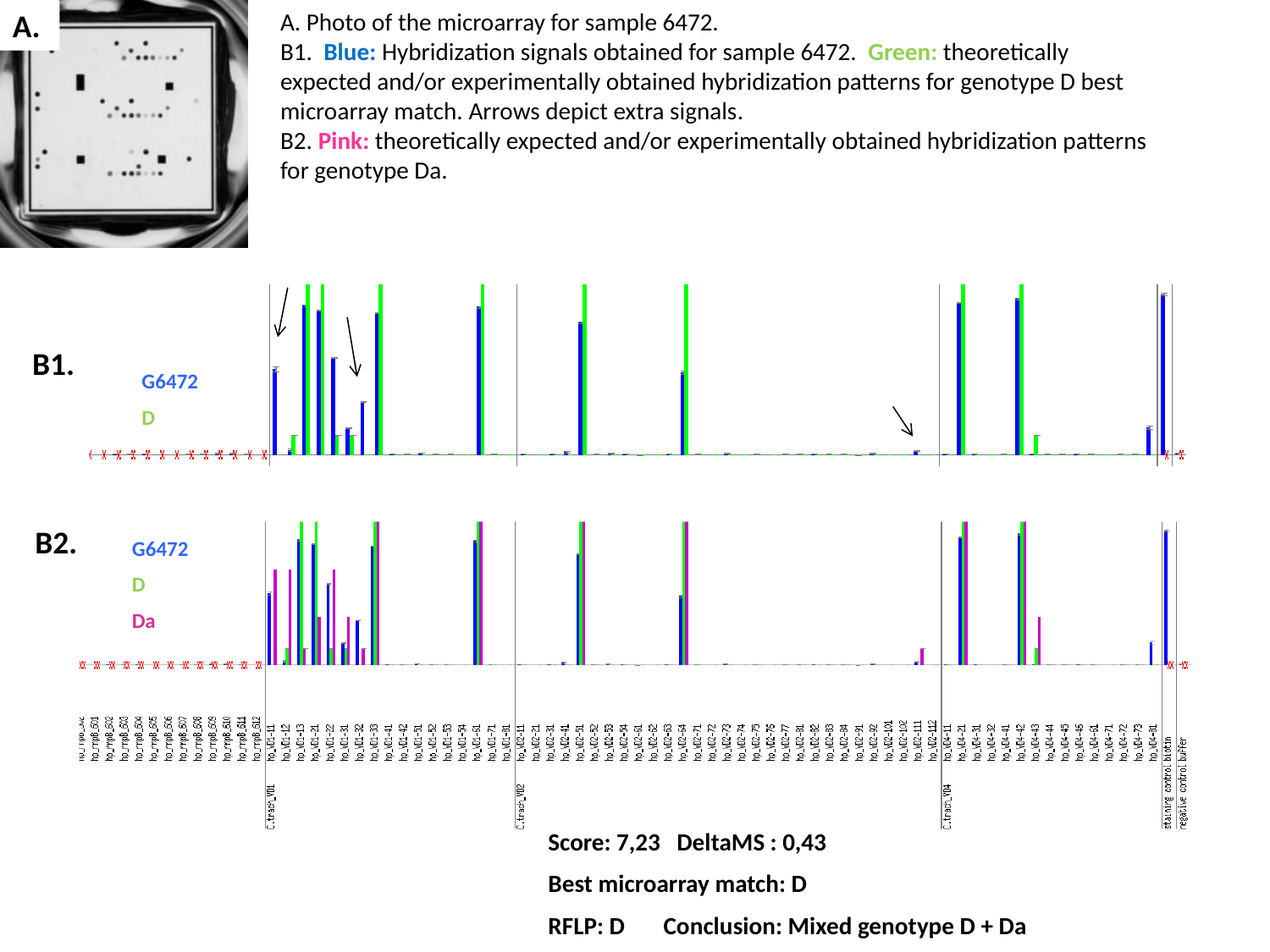

A.
A. Photo of the microarray for sample 6472.
B1. Blue: Hybridization signals obtained for sample 6472. Green: theoretically expected and/or experimentally obtained hybridization patterns for genotype D best microarray match. Arrows depict extra signals.
B2. Pink: theoretically expected and/or experimentally obtained hybridization patterns for genotype Da.
G6472
D
G6472
D
Da
B1.
B2.
Score: 7,23 DeltaMS : 0,43
Best microarray match: D
RFLP: D Conclusion: Mixed genotype D + Da

## Slide 10
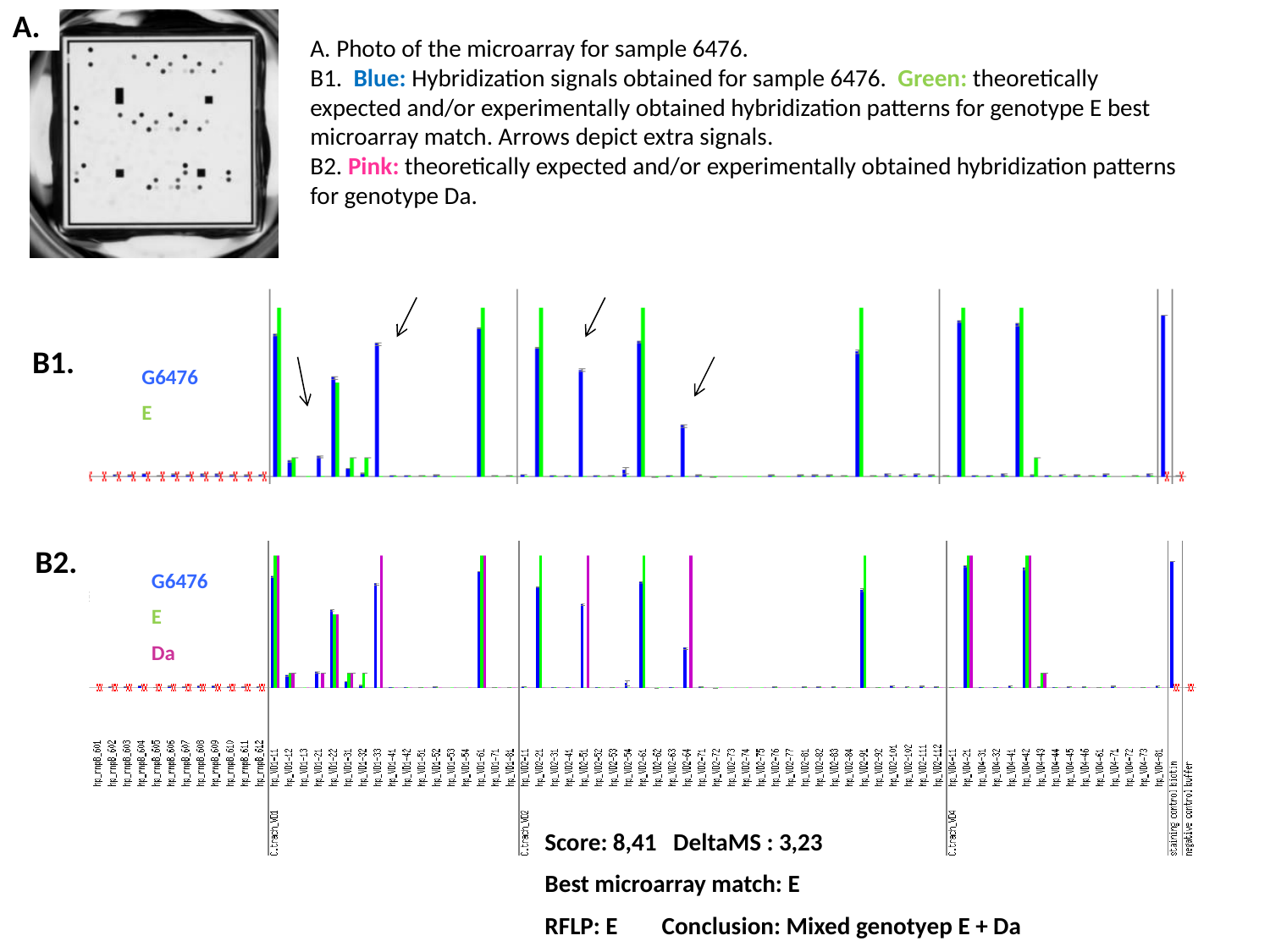

A.
A. Photo of the microarray for sample 6476.
B1. Blue: Hybridization signals obtained for sample 6476. Green: theoretically expected and/or experimentally obtained hybridization patterns for genotype E best microarray match. Arrows depict extra signals.
B2. Pink: theoretically expected and/or experimentally obtained hybridization patterns for genotype Da.
G6476
E
G6476
E
Da
B1.
B2.
Score: 8,41 DeltaMS : 3,23
Best microarray match: E
RFLP: E Conclusion: Mixed genotyep E + Da

## Slide 11
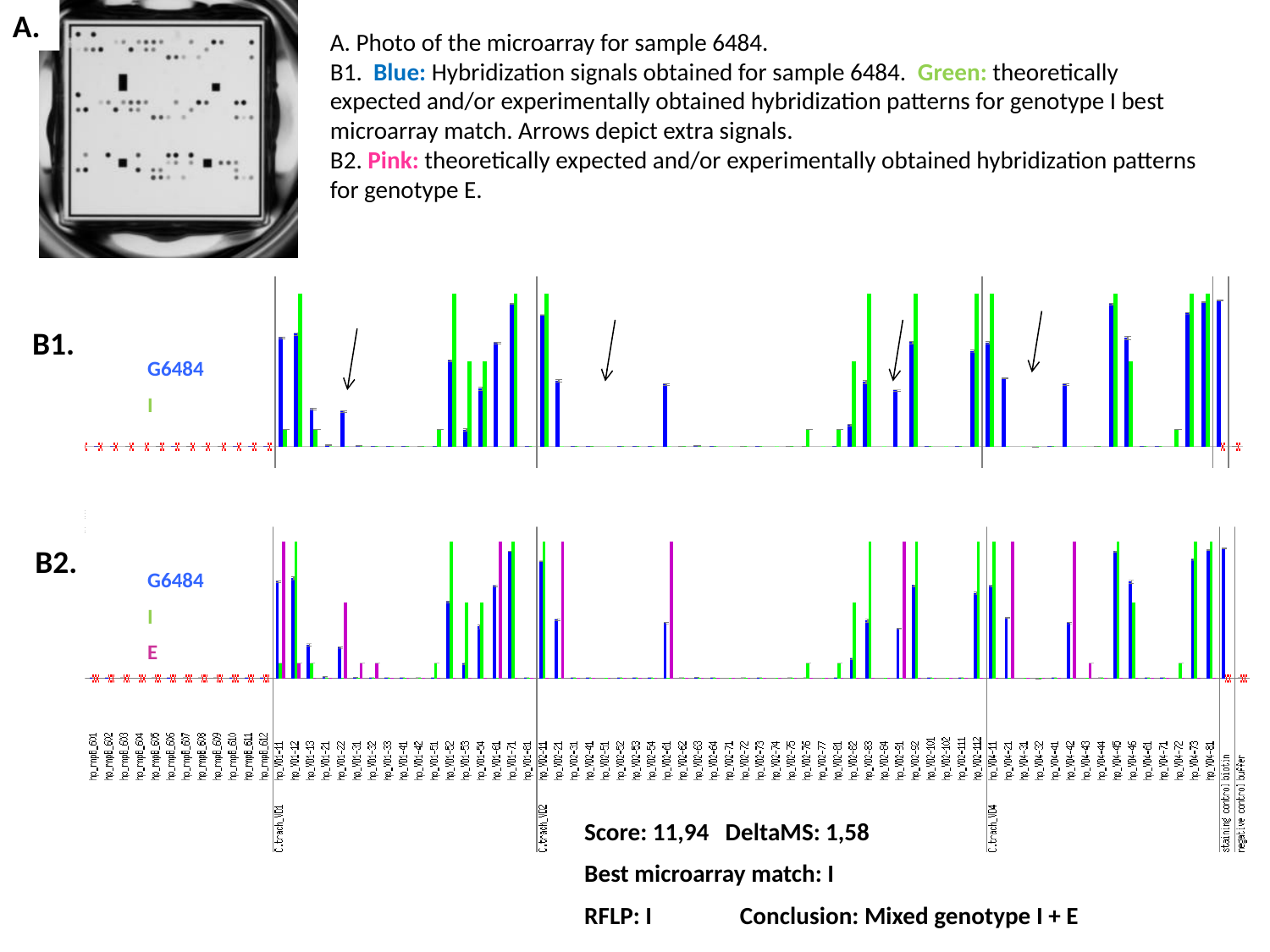

A.
A.
A. Photo of the microarray for sample 6484.
B1. Blue: Hybridization signals obtained for sample 6484. Green: theoretically expected and/or experimentally obtained hybridization patterns for genotype I best microarray match. Arrows depict extra signals.
B2. Pink: theoretically expected and/or experimentally obtained hybridization patterns for genotype E.
G6484
I
G6484
I
E
B1.
B2.
Score: 11,94 DeltaMS: 1,58
Best microarray match: I
RFLP: I Conclusion: Mixed genotype I + E

## Slide 12
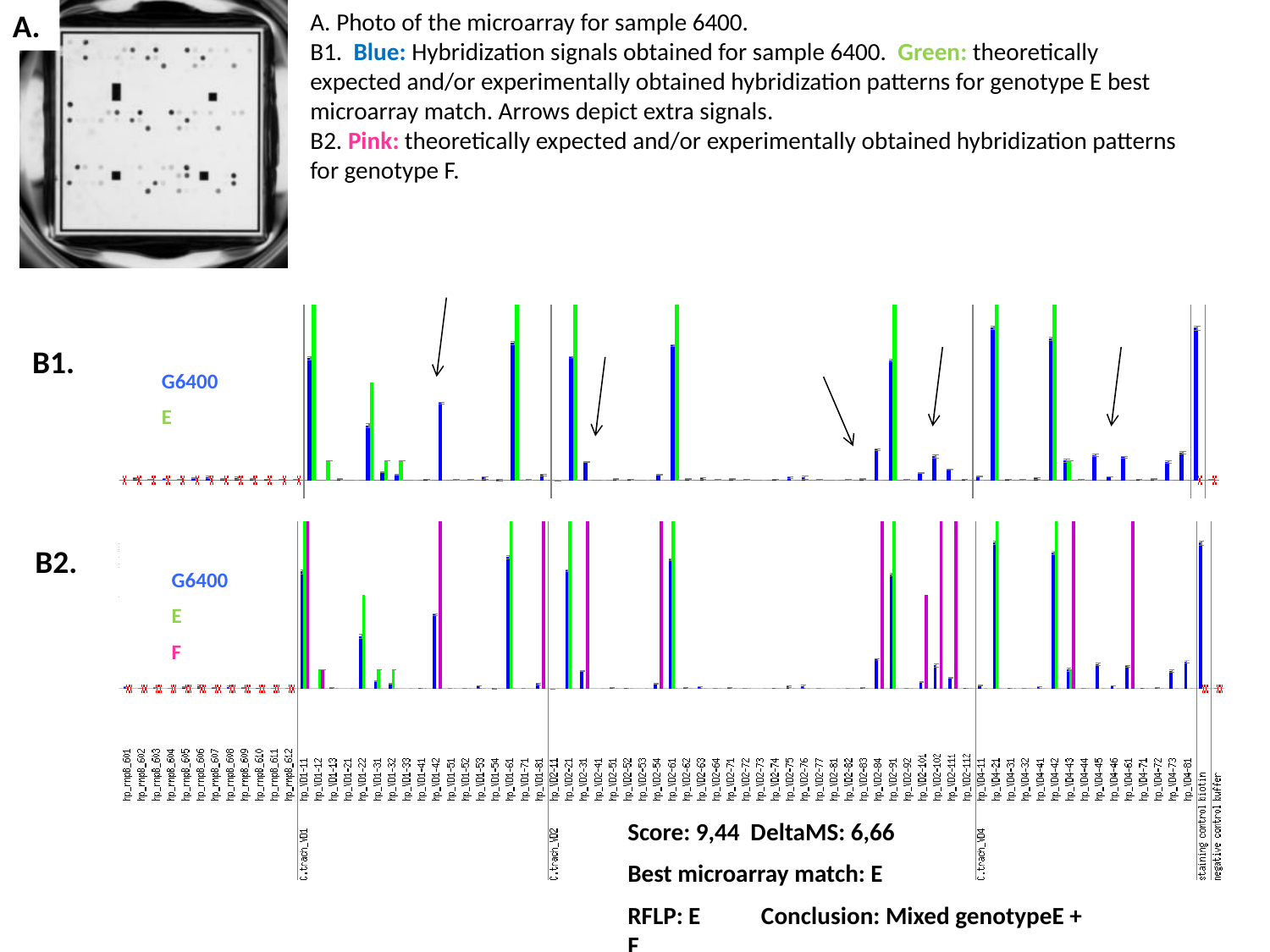

A.
A. Photo of the microarray for sample 6400.
B1. Blue: Hybridization signals obtained for sample 6400. Green: theoretically expected and/or experimentally obtained hybridization patterns for genotype E best microarray match. Arrows depict extra signals.
B2. Pink: theoretically expected and/or experimentally obtained hybridization patterns for genotype F.
G6400
E
G6400
E
F
B1.
B2.
Score: 9,44 DeltaMS: 6,66
Best microarray match: E
RFLP: E Conclusion: Mixed genotypeE + F

## Slide 13
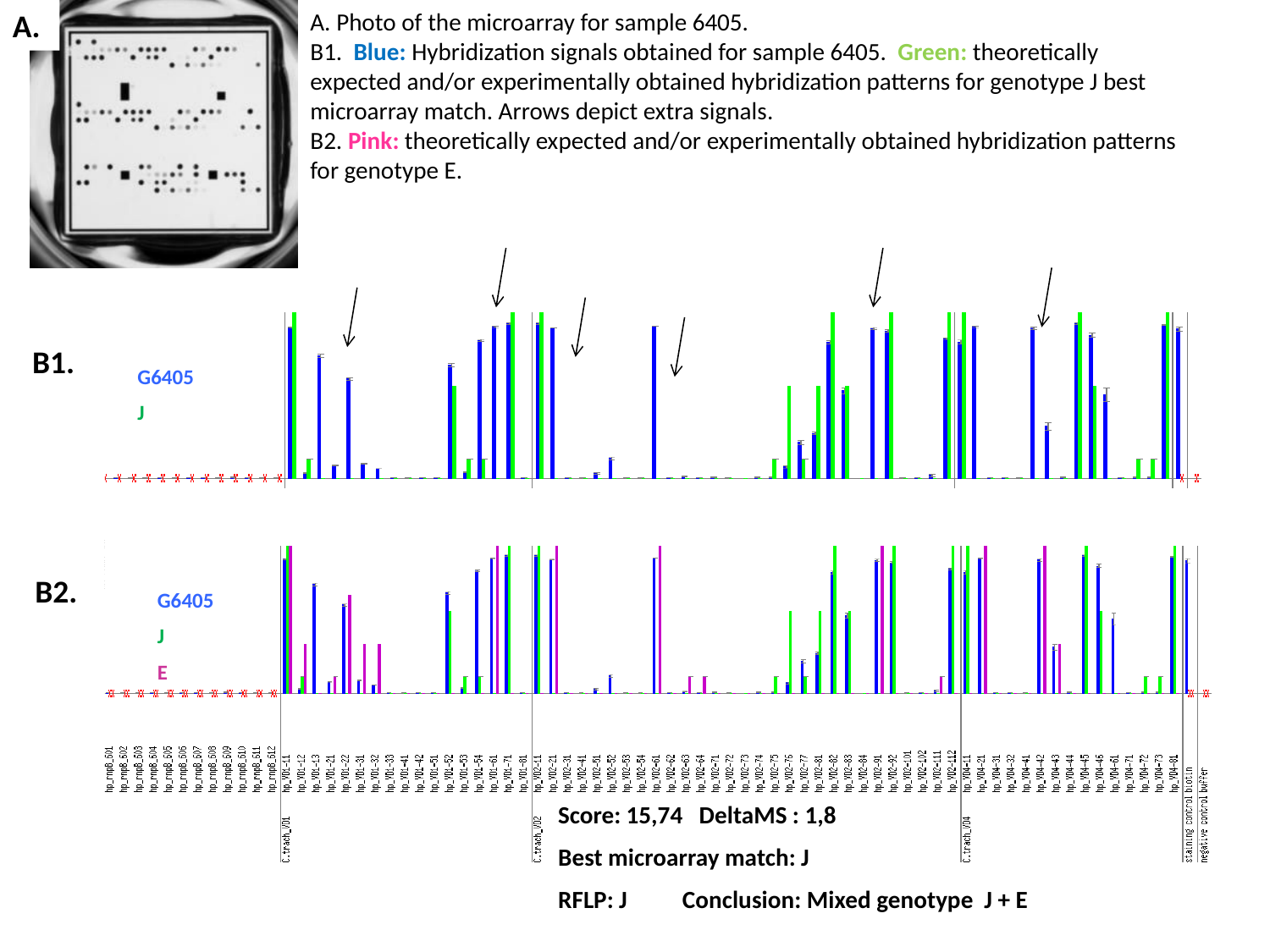

A.
A. Photo of the microarray for sample 6405.
B1. Blue: Hybridization signals obtained for sample 6405. Green: theoretically expected and/or experimentally obtained hybridization patterns for genotype J best microarray match. Arrows depict extra signals.
B2. Pink: theoretically expected and/or experimentally obtained hybridization patterns for genotype E.
G6405
J
G6405
J
E
B1.
B2.
Score: 15,74 DeltaMS : 1,8
Best microarray match: J
RFLP: J Conclusion: Mixed genotype J + E

## Slide 14
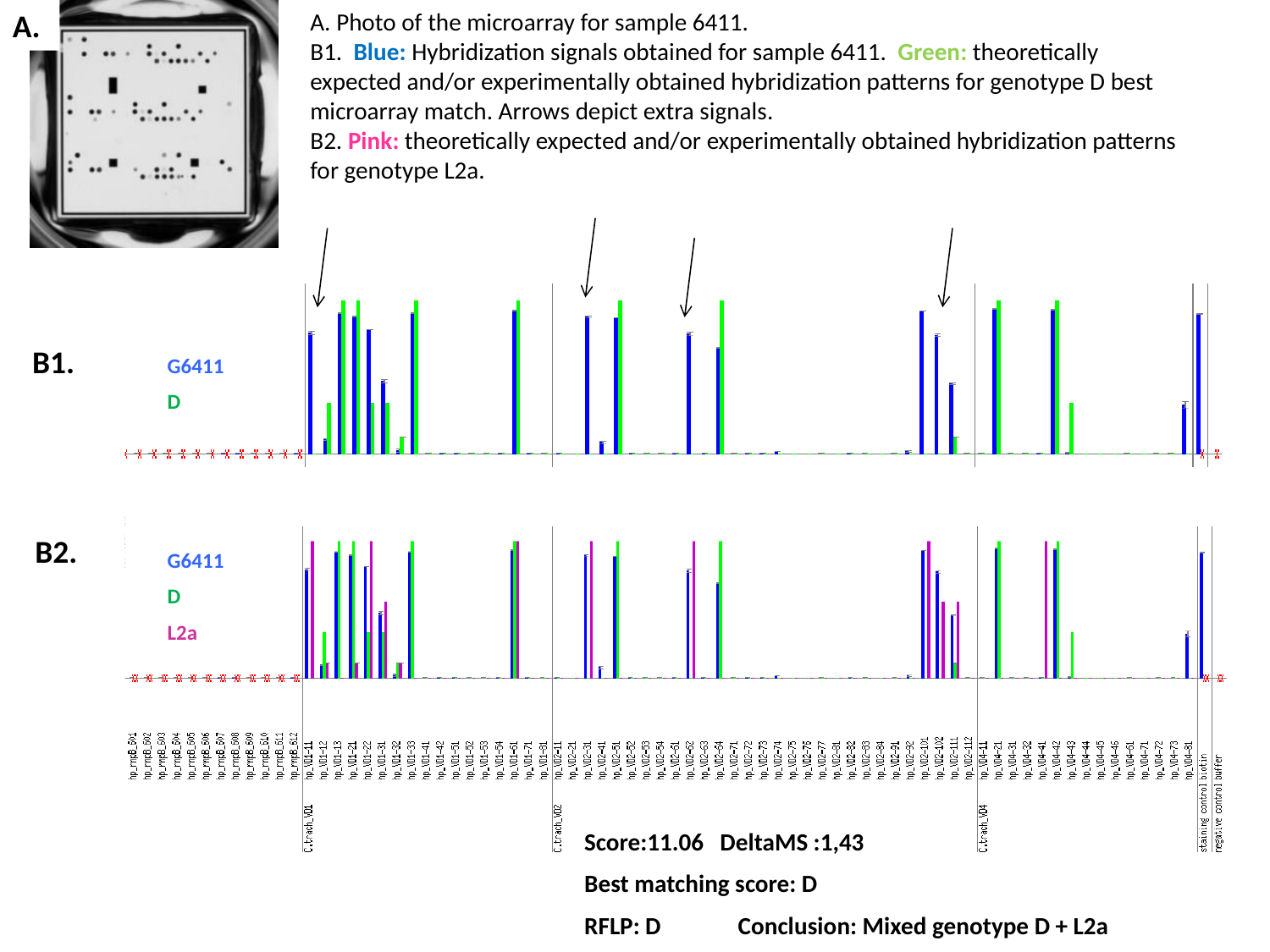

A.
A. Photo of the microarray for sample 6411.
B1. Blue: Hybridization signals obtained for sample 6411. Green: theoretically expected and/or experimentally obtained hybridization patterns for genotype D best microarray match. Arrows depict extra signals.
B2. Pink: theoretically expected and/or experimentally obtained hybridization patterns for genotype L2a.
G6411
D
G6411
D
L2a
B1.
B2.
Score:11.06 DeltaMS :1,43
Best matching score: D
RFLP: D Conclusion: Mixed genotype D + L2a

## Slide 15
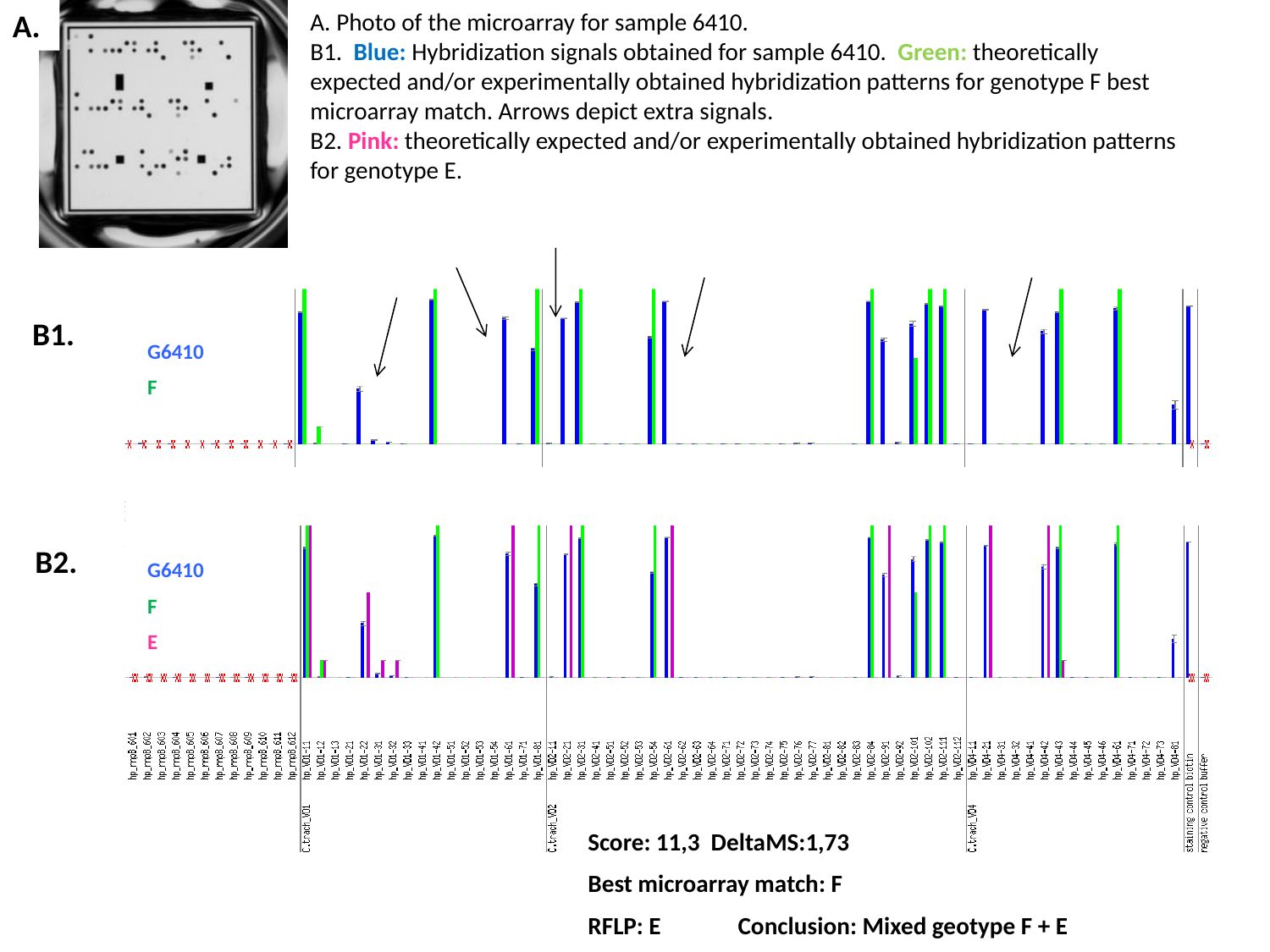

A.
A. Photo of the microarray for sample 6410.
B1. Blue: Hybridization signals obtained for sample 6410. Green: theoretically expected and/or experimentally obtained hybridization patterns for genotype F best microarray match. Arrows depict extra signals.
B2. Pink: theoretically expected and/or experimentally obtained hybridization patterns for genotype E.
G6410
F
G6410
F
E
B1.
B2.
Score: 11,3 DeltaMS:1,73
Best microarray match: F
RFLP: E Conclusion: Mixed geotype F + E

## Slide 16
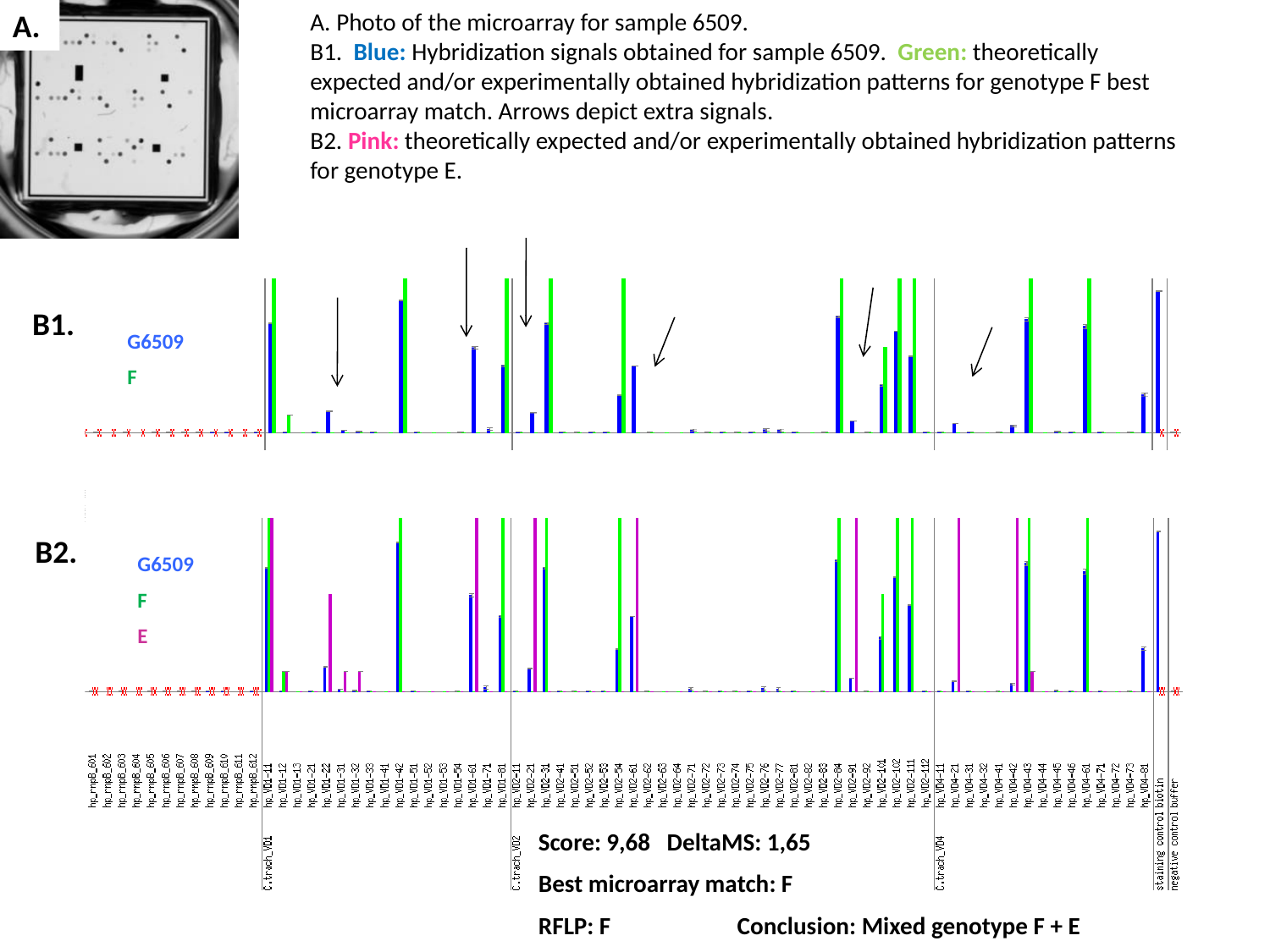

A.
A. Photo of the microarray for sample 6509.
B1. Blue: Hybridization signals obtained for sample 6509. Green: theoretically expected and/or experimentally obtained hybridization patterns for genotype F best microarray match. Arrows depict extra signals.
B2. Pink: theoretically expected and/or experimentally obtained hybridization patterns for genotype E.
G6509
F
G6509
F
E
B1.
B2.
Score: 9,68 DeltaMS: 1,65
Best microarray match: F
RFLP: F Conclusion: Mixed genotype F + E

## Slide 17
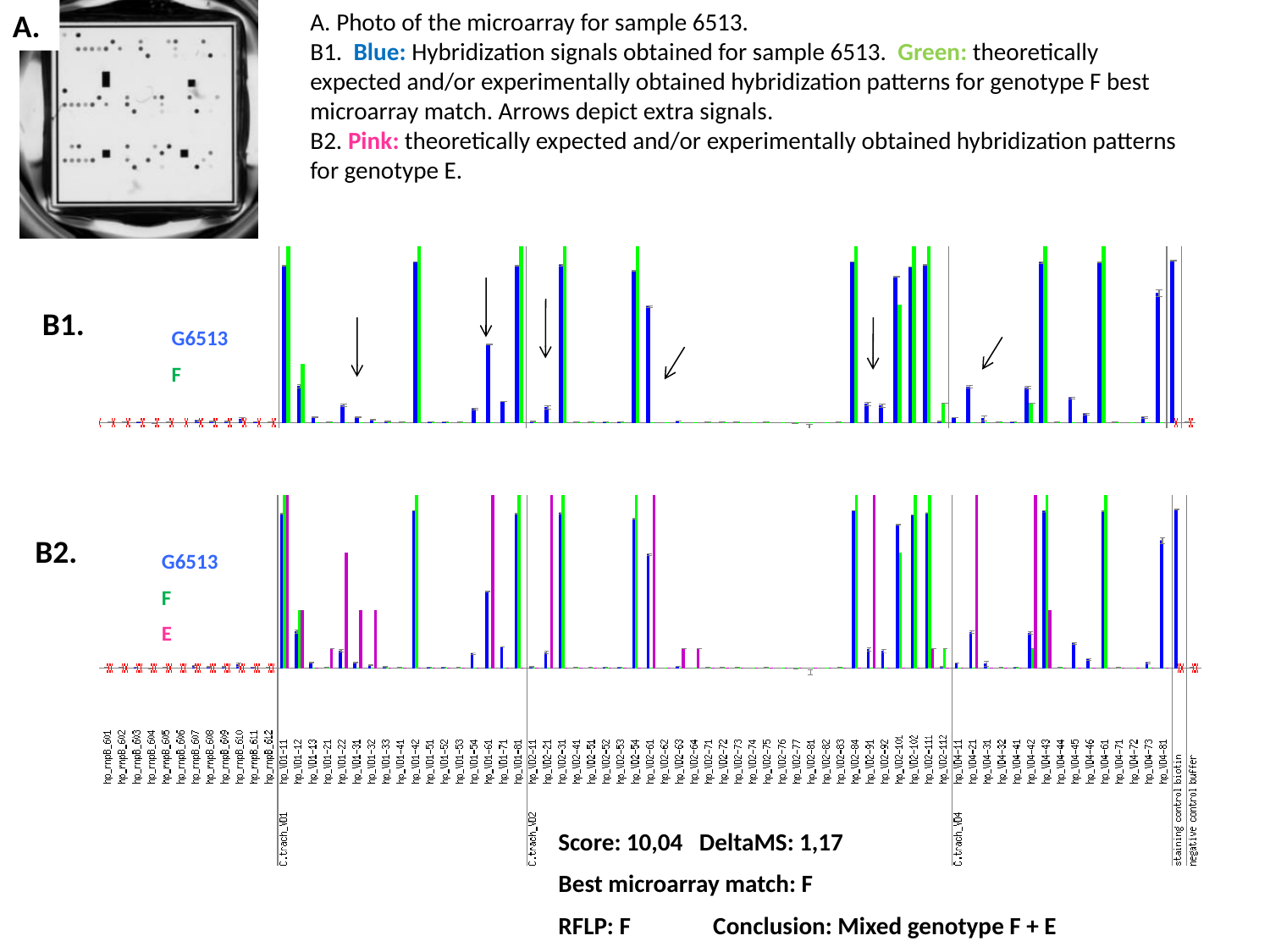

A.
A. Photo of the microarray for sample 6513.
B1. Blue: Hybridization signals obtained for sample 6513. Green: theoretically expected and/or experimentally obtained hybridization patterns for genotype F best microarray match. Arrows depict extra signals.
B2. Pink: theoretically expected and/or experimentally obtained hybridization patterns for genotype E.
G6513
F
G6513
F
E
B1.
B2.
Score: 10,04 DeltaMS: 1,17
Best microarray match: F
RFLP: F Conclusion: Mixed genotype F + E

## Slide 18
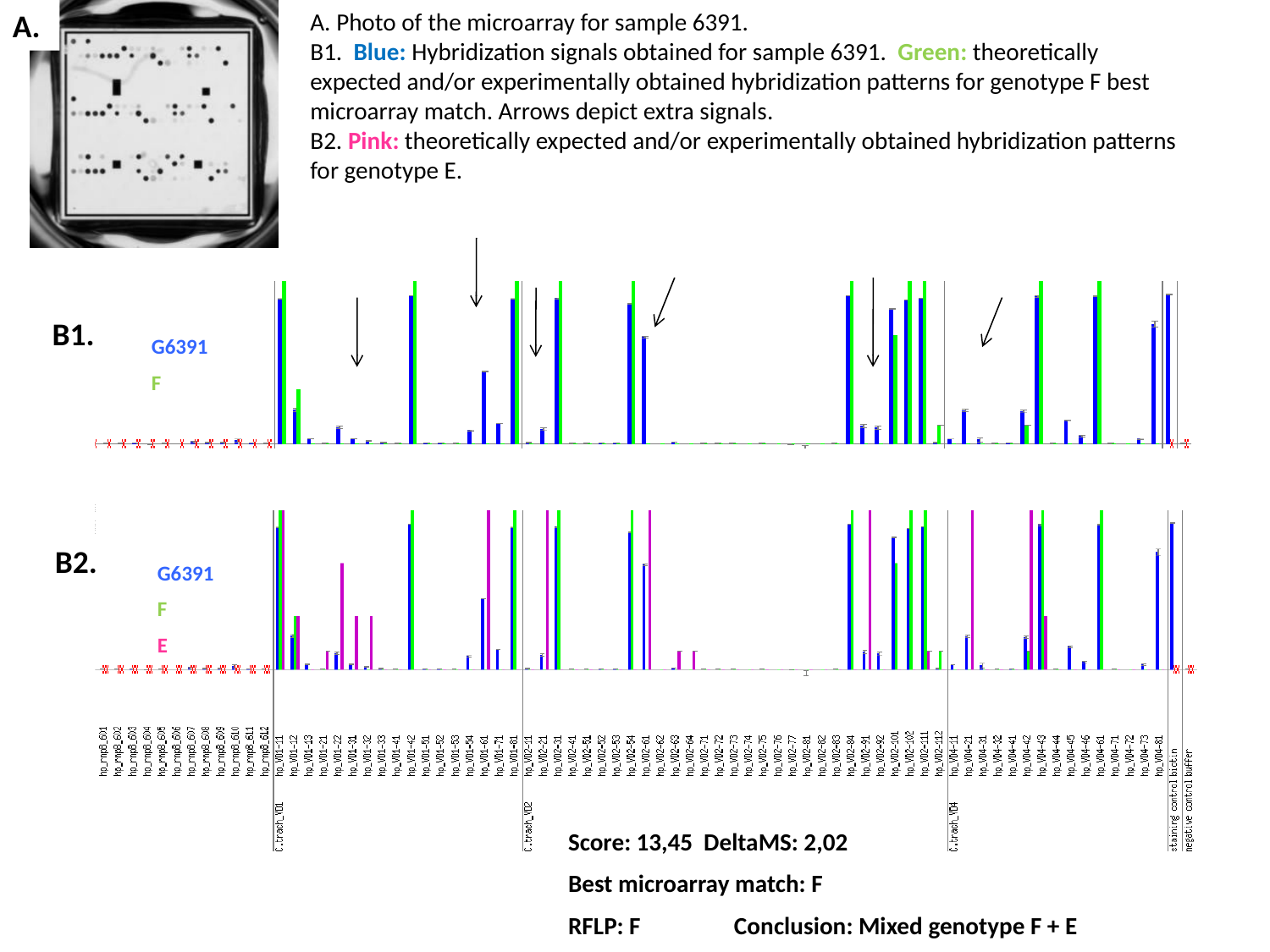

A.
A. Photo of the microarray for sample 6391.
B1. Blue: Hybridization signals obtained for sample 6391. Green: theoretically expected and/or experimentally obtained hybridization patterns for genotype F best microarray match. Arrows depict extra signals.
B2. Pink: theoretically expected and/or experimentally obtained hybridization patterns for genotype E.
G6391
F
G6391
F
E
B1.
B2.
Score: 13,45 DeltaMS: 2,02
Best microarray match: F
RFLP: F Conclusion: Mixed genotype F + E

## Slide 19
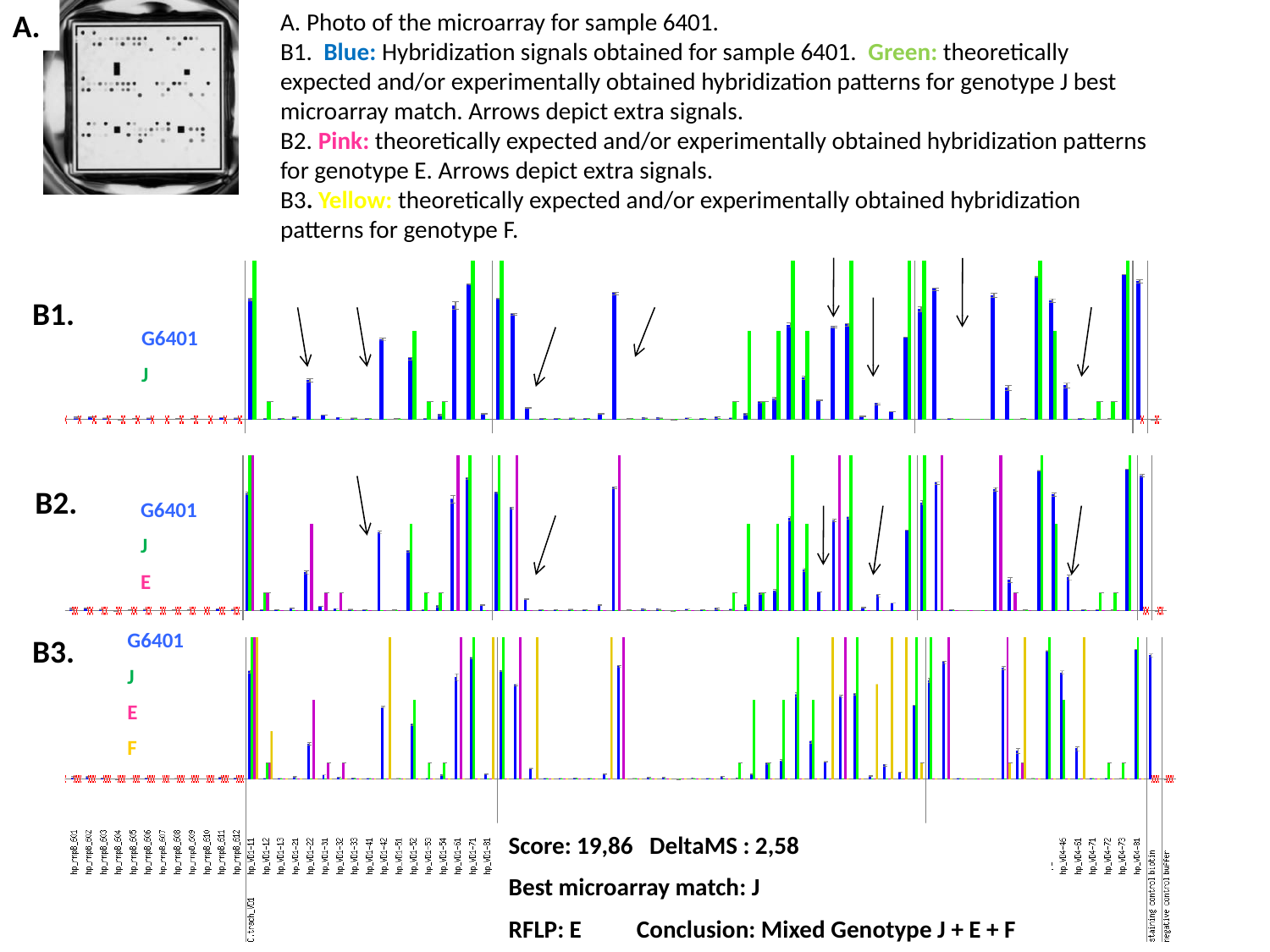

A.
A. Photo of the microarray for sample 6401.
B1. Blue: Hybridization signals obtained for sample 6401. Green: theoretically expected and/or experimentally obtained hybridization patterns for genotype J best microarray match. Arrows depict extra signals.
B2. Pink: theoretically expected and/or experimentally obtained hybridization patterns for genotype E. Arrows depict extra signals.
B3. Yellow: theoretically expected and/or experimentally obtained hybridization patterns for genotype F.
G6401
J
G6401
J
E
G6401
J
E
F
B1.
B2.
B3.
Score: 19,86 DeltaMS : 2,58
Best microarray match: J
RFLP: E Conclusion: Mixed Genotype J + E + F
